# Predicting HER2 overexpression in prostate cancer using machine learning: implications for personalized therapy

**DOI:** 10.3389/fonc.2025.1707946

**Published:** 2026-01-13

**Authors:** Xuantong Huang, Zhen Jiang, Xun Wang, Jie Gao, Danyan Li, Qing Zhang, Xiaozhi Zhao, Hongqian Guo

**Affiliations:** 1 Department of Urology, Nanjing Drum Tower Hospital, The Affiliated Hospital of Nanjing University Medical School, Nanjing, Jiangsu, China; 2 Department of Andrology, Nanjing Drum Tower Hospital, The Affiliated Hospital of Nanjing University Medical School, Nanjing, China; 3 Institute of Urology, Nanjing University, Nanjing, China; 4 Department of Radiology, Nanjing Drum Tower Hospital, The Affiliated Hospital of Nanjing University Medical School, Nanjing, China

**Keywords:** HER2, machine learning, magnetic resonance imaging, prostate cancer, radiomics

## Abstract

**Background:**

Human Epidermal Growth Factor Receptor 2 (HER2), a component of the epidermal growth factor receptor family, is thought to be related to advanced prostate cancer (PCa) when overexpressed. Currently, most research on HER2 is limited to molecular pathology, with relatively few studies focused on imaging aspects.

**Objectives:**

To develop a predictive model by extracting high-throughput radiomics features from magnetic resonance imaging and combining them with clinical characteristics for predicting HER2 overexpression.

**Materials and methods:**

A total of 201 patients who underwent radical prostatectomy and HER2 immunohistochemistry were retrospectively enrolled. These patients were randomly divided into a training set (n=160) and a test set (n=41). Multimodal radiomics features extracted from T2-weighted imaging (T2WI) and apparent diffusion coefficient maps (ADC) were selected using Mann-Whitney U test and least absolute shrinkage and selection operator (LASSO) with ten-fold cross-validation. Predictive models were developed and evaluated based on discrimination and clinical utility.

**Results:**

The combined model integrating ISUP Grade, PSA and Radscore achieved an area under the curve (AUC) of 0.841 (95% CI: 0.697-0.955) in the test set, significantly outperforming the clinical model (AUC = 0.580; *p* = 0.02, DeLong test) and demonstrating a modest improvement over the Radscore model (AUC = 0.838 (0.693-0.951). Evaluation results showed consistent discriminatory power: 0.78 accuracy, 0.77 sensitivity, and 0.79 specificity, indicating well-balanced performance between positive and negative classes. Decision curve analysis and Waterfall plot demonstrated strong clinical applicability.

**Conclusion:**

The combined model effectively predicts HER2 overexpression in prostate cancer, with potential to inform more personalized treatment strategies for HER2-overexpressing PCa patients.

## Introduction

Prostate cancer (PCa) remains one of the most common malignancies in men globally, presenting a substantial public health challenge due to its highly variable clinical behavior and outcomes (1). Although many cases follow an indolent course, a clinically significant subset—characterized by high-grade disease, molecular alterations, or advanced staging—is associated with elevated risks of disease progression, therapeutic resistance, and mortality (2, 3). Among the emerging molecular drivers of aggressive prostate cancer, human epidermal growth factor receptor 2 (HER2) has gained increasing attention for its established roles in tumorigenesis, metastasis, and treatment resistance, as evidenced in cancers such as breast and gastric malignancies (4–6).

In prostate cancer, HER2 overexpression activates key signaling pathways, including PI3K/AKT and MAPK/ERK, which promote tumor cell proliferation, survival, and resistance to androgen deprivation therapy (ADT) (7, 8). Accumulating clinical evidence further associates HER2 overexpression with adverse prognostic outcomes, including shortened progression-free and overall survival, particularly in patients with castration-resistant prostate cancer (CRPC) (9–11). Current assessment of HER2 status relies primarily on immunohistochemistry (IHC)—an invasive, time-consuming approach constrained by tissue heterogeneity, sampling variability, and interobserver disagreement (12, 13). These limitations highlight the pressing need for reproducible, non-invasive methods for HER2 phenotyping.

Recent advances in radiomics and artificial intelligence (AI) have opened promising avenues for non-invasive biomarker discovery. As demonstrated by Piras et al., AI-integrated predictive models are increasingly being applied in prostate cancer to optimize treatment personalization, such as predicting radiotherapy-related toxicity using dosimetric and imaging data (14). Similarly, Sekhoacha et al. and Varaprasad et al. have outlined the expanding role of genetic profiling and novel diagnostic modalities—including serum, urine, and tissue biomarkers—in refining risk stratification and therapeutic decision-making for prostate cancer patients (13, 15). These developments reflect a broader shift toward precision oncology, in which imaging-derived biomarkers can effectively complement molecular assays. In this context, HER2 has re-emerged as an actionable therapeutic target. The recent U.S. FDA approval of trastuzumab deruxtecan (T-DXd) for HER2-positive solid tumors represents a pivotal advancement, creating new opportunities for targeted therapy in HER2-overexpressing prostate cancer (16–19). Despite this therapeutic potential, the non-invasive detection of HER2 status via radiomics remains underexplored. Therefore, this study aims to develop and validate an interpretable machine learning model for the non-invasive prediction of HER2 overexpression in prostate cancer by integrating radiomic features derived from biparametric MRI with clinical variables. Our work seeks to contribute to the growing paradigm of image-based molecular phenotyping and support personalized treatment planning in oncology.

## Materials and methods

### Study design and participants

**Figure 1:**
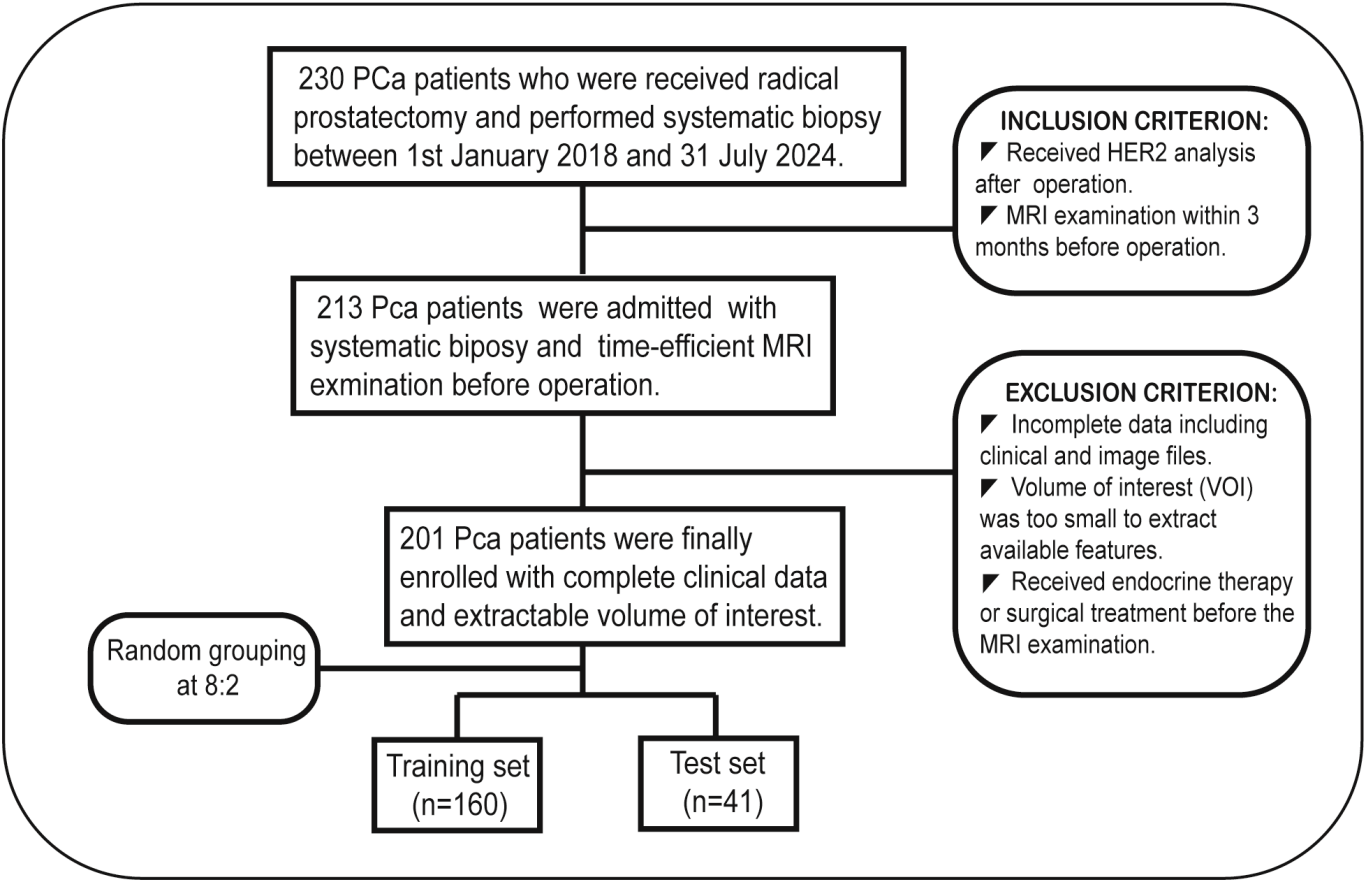
Flowchart of participant inclusion and exclusion.

This study enrolled patients who underwent radical prostatectomy and had HER2 immunohistochemistry performed on the resected specimens between January 1, 2018, and July 31, 2024, at our hospital. All preoperative clinical and imaging data for the patients were collected within three months. Patients who had undergone other treatment modalities were selectively excluded. The pathological results are assessed based on the immunohistochemical findings of the postoperative radical prostatectomy specimen. The detailed inclusion and exclusion criteria are listed as follows (**Figure 1**). Then, 201 selected patients were randomly divided into two groups: a training set (n=160) and a test set (n=41). To prevent data imbalance, the random allocation ensured consistency in the ratio of positive to negative cases between the training and test set.

### Inclusion criteria

Patients diagnosed with prostate cancer and underwent radical prostatectomy.

Immunohistochemical analysis for HER2 was performed on the resected specimens.

Prostate MR images and clinical indicators obtained within three months prior to surgery.

### Exclusion criteria

Patients who received endocrine therapy or surgical treatment before the MRI examination.

Volume of interest (VOI) was too small to extract viable features from the MR image.

Patients with incomplete clinical or imaging data.

### Preoperative clinical parameters

Comprehensive clinical data were prospectively collected prior to surgical intervention. The following parameters were included:

- Age: At the time of surgery.
- Prostate-Specific Antigen (PSA): Serum level measured within one month before surgery.
- Prostate Volume: Determined by transrectal ultrasound.
- PSA Density (PSAD): Calculated as PSA divided by prostate volume.
- Lesion Dimension On MRI: Maximum tumor diameter on preoperative MRI.
- Tumor Focality: Assessed on MRI as unifocal or multifocal.
- Positive Biopsy Cores/Total Cores: Number of positive cores divided by the total number of cores.
- Biopsy ISUP Grade Group: Based on preoperative biopsy pathology.
- PI-RADS Score (v2.1): Assessed by an expert radiologist with over 10 years of experience.

### Postoperative pathological assessment

Pathological evaluation was performed on whole-mount sections of radical prostatectomy specimens. The following key parameters were recorded:

- ISUP Grade Group (RP): The final grade group assigned after surgical pathology review.
- Pathological T Stage (pT Stage): Extent of primary tumor invasion.
- Pathological N Stage (pN Stage): Lymph node involvement status.

### HER2 overexpression evaluation

**Figure 2:**
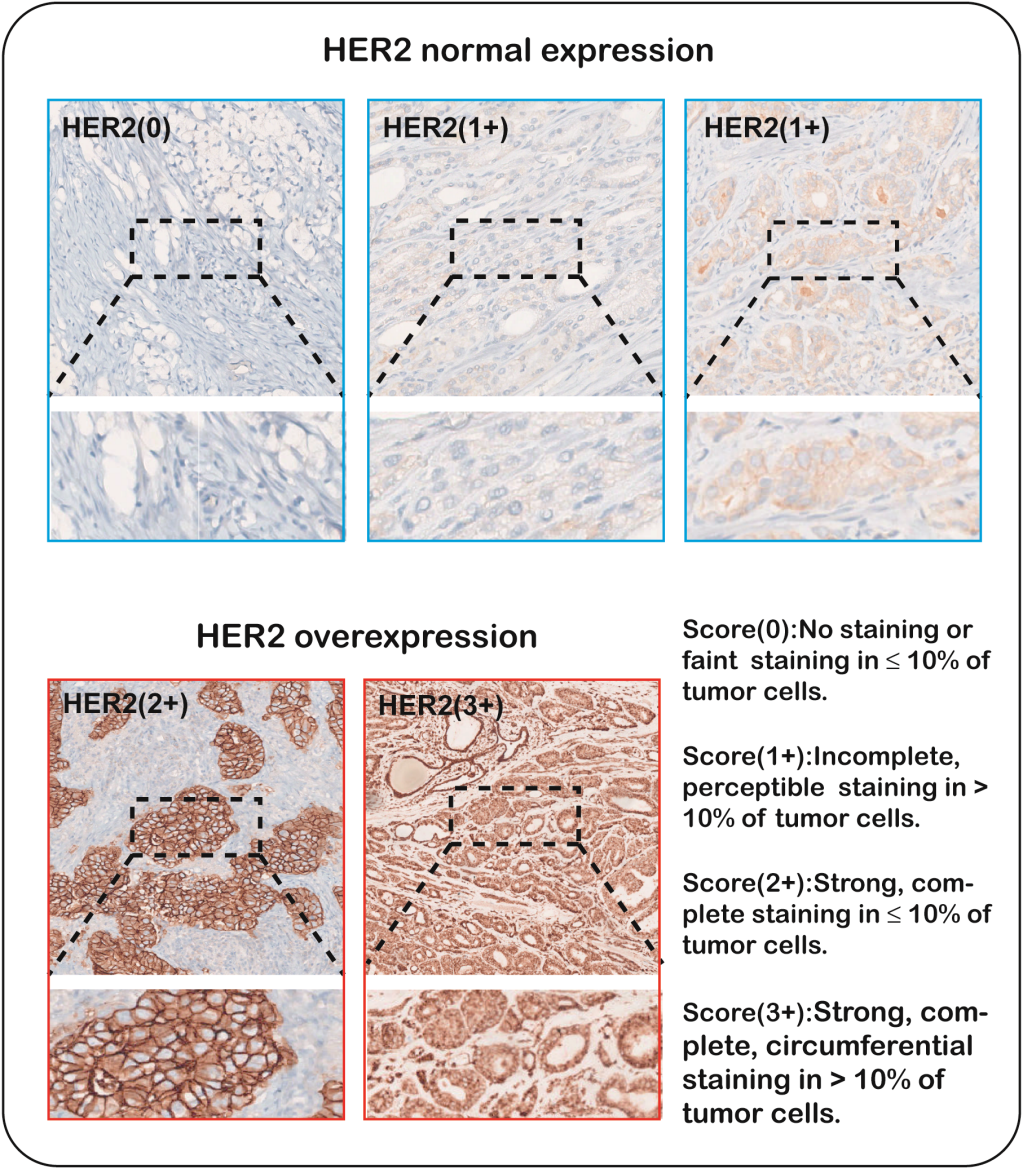
Immunohistochemical scoring of HER2 in prostate cancer under 40xmagnification.

Currently there is no universally accepted international standard for determining HER2 overexpression in prostate cancer. Based on existing IHC criteria and reports from relevant literature, only 20-25% of prostate cancer cases demonstrate moderate HER2 expression (2+), while strong expression (3+) is observed in approximately 5% of cases. A majority (70%-75%) of prostate cancers lack or have low expression of HER2. In normal prostate tissue, HER2 expression is detected in fewer than 5% of cases (12, 13). To address this, we classified HER2 overexpression based on IHC results, defining cases with scores of 2+ to 3+ as overexpression, scores of 0 or 1+ were considered normal expression (no or low expression). All HER2 expression statuses were assessed using specific antibodies on postoperative pathological specimens (**Figure 2**).

### Image segmentation and radiomics feature extraction

**Figure 3:**
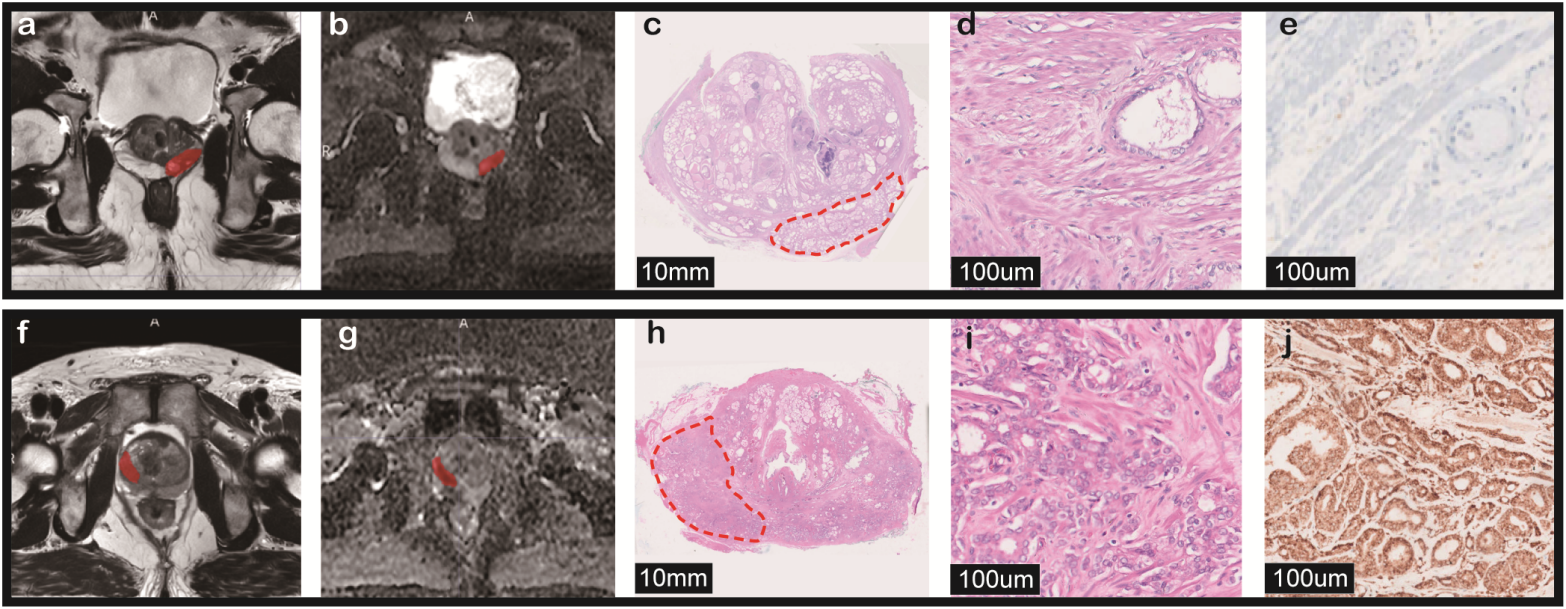
The figures above illustrate axial magnetic resonance imaging (T2WI, ADC) of two cases, along with their corresponding HE staining and immunohistochemical staining. Scanning electron microscopy (SEM) examination of pathological sections was performed at 100x magnification following standard conductive preparation (10mm-100um). Case1: An 81-year-old male presented with an initial PSA level of 6.08 ng/mL. Magnetic resonance imaging (MRI) revealed an abnormal signal in the left peripheral zone of the prostatic mid-gland, with a PI-RADS score of 3. Both preoperative needle biopsy and postoperative pathological sections indicated a Gleason score of 3 + 3 = 6. **(a, b)** Tumor region outlined for feature extraction. **(c, d)** Schematic representation of the postoperative pathological section. **(e)** Immunohistochemistry (IHC) showed HER2 (0). Case 2: A 79-year-old male had a PSA level of 8.77 ng/mL. MRI demonstrated an abnormal signal in the right peripheral zone of the prostatic mid-gland, with a PI-RADS score of 5. Both preoperative needle biopsy and postoperative pathological sections indicated a Gleason score of 4 + 5 = 9. **(f, g)** Tumor region outlined for feature extraction. **(h–i)** Schematic representation of the postoperative pathological section. **(j)** IHC results showed HER2 (3+).

The non-enhanced and axial MRI images at 3.0T, with a thickness of 3mm, were acquired from two sequences (T2WI and ADC) for image segmentation and feature extraction, including a GE Signa HD, a GE 750 Discovery, and a Siemens Skyra, with the following shared parameters: slice thickness: 3 mm; pixel spacing: 0.49 mm; magnetic field strength: 3.0 T; and number of slices: 24. A radiologist and a urologist manually segmented the volume of interest (VOI) along the tumor contour on the axial MRI slices (**Figure 3**). Before feature extraction, all the mask images were normalized with a bin width of 25, followed by N4 bias field correction, and resampled to a pixel spacing of (3, 3, 3).

Then, we got 1,694 high-throughput imaging features from each MRI sequence (847 from T2WI and 847 from ADC), including the original images, Gaussian Laplacian filtering (LoG), and wavelet transform (wavelet) images: shape-based features (n=10), first-order features (n=18), texture features (n=75), LoG and wavelet features (n=744). Texture features included gray-level co-occurrence matrix (GLCM), gray-level size zone matrix (GLSZM), gray-level run length matrix (GLRLM), neighboring gray tone difference matrix (NGTDM), and gray-level dependence matrix (GLDM) features. To ensure good consistency between measurements from different observers, we set an intraclass correlation coefficient (ICC) threshold of > 0.75 for feature filtering.

Supplements: For VOI segmentation, ITK-SNAP was used to mask the tumor area in each sequence. PyRadiomics v3.8 was employed to help radiomics feature extraction. The overall extraction process was conducted in accordance with the guidelines of the Image Biomarker Standardization Initiative.

### Feature preprocessing and feature selection

To mitigate the influence of varying scales across imaging features, all such data were standardized using z-score normalization. Feature selection proceeded in a two-stage approach exclusively on the training cohort. Initially, univariate screening was conducted with the Mann–Whitney test (p < 0.05) to retain features exhibiting significant intergroup differences. These selected features were subsequently subjected to the least absolute shrinkage and selection operator (LASSO) regression with 10-fold cross-validation to further refine the feature set and mitigate multicollinearity. In parallel, univariate logistic regression was applied to clinical variables to identify those significantly associated with the outcome. This integrated process yielded a final set of features most predictive of HER2 overexpression from both imaging and clinical domains. Crucially, the test set remained entirely isolated from all steps of feature selection to prevent data leakage and ensure unbiased model evaluation.

### Model construction and development

Three machine learning classifiers—Random Forest (RF), Support Vector Machine (SVM), and Logistic Regression (LR)—were implemented following feature selection to develop prediction models. Each model underwent training followed by evaluation on a held-out test set. Performance was quantified across multiple metrics: the area under the receiver operating characteristic curve (AUC), accuracy, precision, recall, F1-score, sensitivity, and specificity. The clinical utility of the models was further compared using decision curve analysis (DCA). Additionally, during the model training process on the training set, we employed the SMOTE function to balance the disparity between positive and negative samples, thereby enhancing the stability of the model.

### Statistical analysis

Statistical analyses were conducted using SPSS version 27.0 and Python 3.0. In the analysis of baseline data comparison. For continuous variables, the Mann-Whitney U test was employed; for categorical variables, the Chi-square test or Fisher’s exact test was used, as appropriate. Feature selection and modeling are primarily handled by Python.

### Ethical considerations

This retrospective study was approved by the Hospital Ethics Committee. Given the retrospective nature of the study and the use of de-identified patient data, the requirement for informed consent was waived by the IRB. Informed consent was obtained from all participants prior to their inclusion in the study. Furthermore, anonymization was performed on all patient data. All procedures were conducted in accordance with the ethical standards of the institutional and national research committees, as well as the 1964 Helsinki Declaration and its later amendments, or other comparable ethical guidelines.

In summary, the comprehensive collection and rigorous assessment of clinical data in this study provide a robust foundation for analyzing the prognostic value of radiomics and clinical factors in predicting HER2 overexpression in prostate cancer. The methodology ensures high-quality data and reliable results, contributing to the advancement of precision medicine in prostate cancer management.

## Results

### Baseline characteristics

A total of 201 patients diagnosed with prostate cancer who underwent systematic biopsy after surgical treatment were included in the study. Immunohistochemistry results showed that 33.3% of specimens (n=66) were HER2 overexpression, while 66.7% of specimens (n=135) were HER2 no or low expression. Baseline characteristics of the total cohort are summarized in **Table 1**.

**Table 1:** Patient characteristics in total set.

Characteristic	n=201	HER2 Status		P-value
		No or low expression (n=135)	Overexpression (n=66)	
Age (y)	71.00 (67.00-75.00)	72 (67.00-75.50)	71.00 (65.25-75.00)	0.408
PSA level (ng/ml)	10.79 (7.09-18.00)	10.30 (6.67-15.10)	11.40 (8.11-20.59)	0.067
Prostate Volume (ml)	31.70 (25.90-44.00)	30.90 (24.85-43.74)	35.25 (27.88-46.88)	0.161
PSAD (ng/ml/ml)	0.31 (0.21-0.52)	0.30 (0.20-0.48)	0.33 (0.22-0.58)	0.261
Lesion Dimension On MRI (cm)	2.10 (1.50-2.70)	2.00 (1.50-2.60)	2.20 (1.52-2.95)	0.254
Positive Biopsy Cores/Total Cores	0.36 (0.25-0.50)	0.36 (0.25-0.50)	0.36 (0.25-0.66)	0.319
Biopsy ISUP Grade Group				0.150
1	37 (18.4%)	28 (20.7%)	9 (13.6%)	
2	63 (31.3%)	46 (34.1%)	17 (25.8%)	
3	54 (26.9%)	35 (25.9%)	19 (28.8%)	
≥4	47 (23.4%)	26 (19.3%)	21 (31.8%)	
PI-RADS Score				0.755
3	40 (19.9%)	28 (20.7%)	12 (18.2%)	
4	83 (41.3%)	57 (42.2%)	26 (39.4%)	
5	78 (38.8%)	50 (37.0%)	28 (42.4%)	
Tumor Focality				0.457
Unifocal	105 (52.2%)	68 (50.4%)	37 (56.1%)	
Multifocal	96 (47.8%)	67 (49.6%)	29 (43.9%)	
ISUP Grade Group (RP)				0.024
1	12 (6.0%)	8 (5.9%)	4 (6.1%)	
2	95 (47.3%)	72 (53.3%)	23 (34.8%)	
3	73 (36.3%)	46 (34.1%)	27 (40.9%)	
≥4	21 (10.4%)	9 (6.7%)	12 (18.2%)	
pT Stage				0.927
2	94 (46.8%)	62 (45.9%)	32 (48.5%)	
3a	87 (43.3%)	59 (43.7%)	28 (42.4%)	
3b	20 (10.0%)	14 (10.4%)	6 (9.1%)	
pN Stage				1.000
0	187 (93.0%)	125 (92.6%)	62 (93.9%)	
1	14 (7.0%)	10 (7.4%)	4 (6.1%)	

1. Continuous variables are presented as median (Q1-Q3). 2. Categorical variables are presented as count (percentage). 3. P-values < 0.05 are considered statistically significant. 4. Fisher’s exact test for 2×2 categorical variables. 5. Chi-square test for multi-category categorical variables. 6. HER2, Human epidermal growth factor receptor 2; PSA, prostate-specific antigen; PSAD, prostate- specific antigen density; MRI, magnetic resonance imaging; ISUP Grade, International Society of Urological Pathology Grade; PI-RADS, Prostate Imaging Reporting and Data System; RP, radical prostatectomy.

Twenty percent of the patients were randomly assigned to test set. In the training set (n=160), 53 (33.1%) patients were HER2 overexpression, and 107 (66.9%) patients were HER2 no or low expression. In the test set (n=41), 13 (31.7%) patients were HER2 overexpression, and 28 (68.3%) patients were HER2 no or low expression. **Table 2** summarizes the baseline characteristics of patients in the training and test cohorts.

**Table 2:** Baseline patient characteristics between the training and test sets.

Baseline characteristic	Total set (n=201)	Training set (n=160)	Test set (n=41)	P-value
Age (y)	71.00 (67.00-75.00)	71.00 (66.75-76.00)	72.00 (68.00-75.00)	0.874
PSA level (ng/ml)	10.79 (7.09-18.00)	10.25 (6.91-16.48)	12.30 (7.94-20.60)	0.245
Prostate Volume (ml)	31.70 (25.90-44.00)	32.15 (26.40-45.23)	29.40 (24.70-41.20)	0.327
PSAD (ng/ml/ml)	0.31 (0.21-0.52)	0.30 (0.21-0.50)	0.37 (0.21-0.55)	0.179
Lesion Dimension On MRI (cm)	2.10 (1.50-2.70)	2.10 (1.50-2.70)	2.10 (1.40-2.70)	0.841
Positive Biopsy Cores/Total Cores	0.36 (0.25-0.50)	0.36 (0.25-0.50)	0.33 (0.25-0.50)	0.974
Biopsy ISUP Grade Group				0.882
1	37 (18.4%)	30 (18.8%)	7 (17.1%)	
2	63 (31.3%)	48 (30.0%)	15 (36.6%)	
3	54 (26.9%)	44 (27.5%)	10 (24.4%)	
≥4	47 (23.4%)	38 (23.8%)	9 (22.0%)	
PI-RADS Score				0.998
3	40 (19.9%)	32 (20.0%)	8 (19.5%)	
4	83 (41.3%)	66 (41.2%)	17 (41.5%)	
5	78 (38.8%)	62 (38.8%)	16 (39.0%)	
Tumor Focality				0.604
Unifocal	105 (52.2%)	82 (51.2%)	23 (56.1%)	
Multifocal	96 (47.8%)	78 (48.8%)	18 (43.9%)	
ISUP Grade Group (RP)				0.736
1	12 (6.0%)	11 (6.9%)	1 (2.4%)	
2	95 (47.3%)	74 (46.2%)	21 (51.2%)	
3	73 (36.3%)	58 (36.2%)	15 (36.6%)	
≥4	21 (10.4%)	17 (10.6%)	4 (9.8%)	
pT Stage				0.711
2	94 (46.8%)	77 (48.1%)	17 (41.5%)	
3a	87 (43.3%)	68 (42.5%)	19 (46.3%)	
3b	20 (10.0%)	15 (9.4%)	5 (12.2%)	
pN Stage				0.490
0	187 (93.0%)	150 (93.8%)	37 (90.2%)	
1	14 (7.0%)	10 (6.2%)	4 (9.8%)	
HER2 Status				1.000
No or low expression	135 (67.2%)	107 (66.9%)	28 (68.3%)	
Overexpression	66 (32.8%)	53 (33.1%)	13 (31.7%)	

No significant difference in performance was found between the training and test sets. PSA = prostate-specific antigen; PSAD, prostate- specific antigen density; MRI, magnetic resonance imaging; ISUP Grade, International Society of Urological Pathology Grade; PI-RADS, Prostate Imaging Reporting and Data System; RP, radical prostatectomy; HER2, Human epidermal growth factor receptor 2.

### Feature selection and model construction

**Figure 4:**
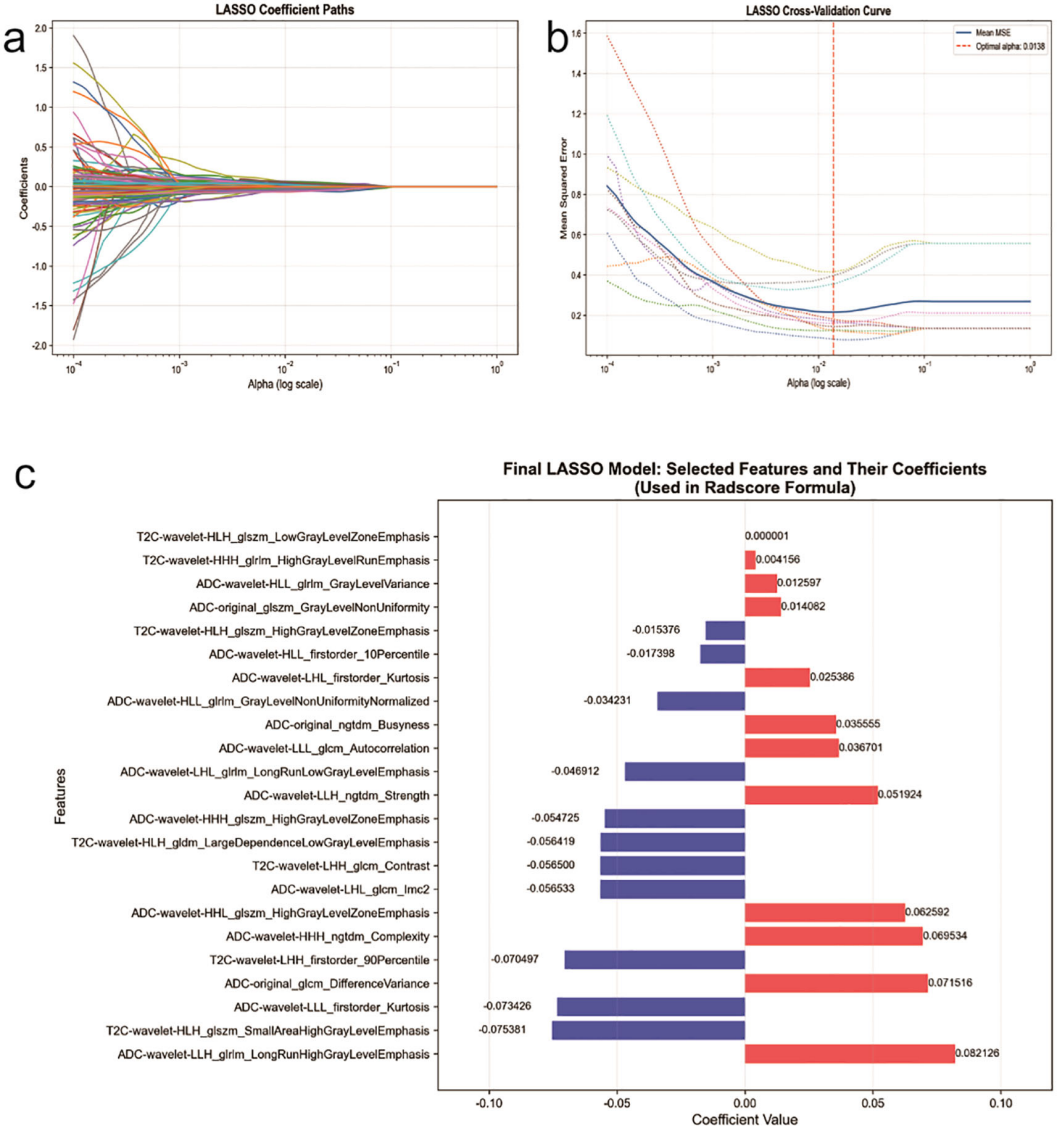
Radiomics feature screening engineering flowchart. **(a)** Lasso coefficient path plot. **(b)** Lasso path plot mean squared error plot. **(c)** Variable selection trace.

Radiomic feature stability was assessed using the intraclass correlation coefficient. Based on an ICC threshold of > 0.75, 1257 robust features were selected from the original 1694 for subsequent analysis, ensuring reproducibility. Feature selection was then performed on the training cohort in a two-stage approach. First, the Mann-Whitney U test (p < 0.05) was applied to identify features with significant differences between groups. Second, the LASSO regression with 10-fold cross-validation was employed to further reduce redundancy and select the most predictive features. This process resulted in the final selection of 23 non-redundant and informative features, as visualized in the selection pathway (**Figure 4**). A radiomics score (Radscore) was computed for each patient as a linear combination of the selected features weighted by their respective LASSO coefficients. The detailed calculation formula is provided in the **Supplementary Materials**. Finally, the Radscore was incorporated into a logistic regression model to predict HER2 overexpression. The model demonstrated strong discriminatory performance, achieving an AUC of 0.838 in the test set.

Concurrently, univariable logistic regression analysis was performed on preoperative clinical variables within the training set to identify potential predictors, using a liberal significance threshold of *p* < 0.10 to avoid overlooking marginally significant features (**Table 3**). Two clinical variables were subsequently retained for model construction: PSA level and ISUP Grade Group (dichotomized as 3–5 versus 1-2). A clinical model based solely on these two predictors was developed using logistic regression, yielding an area under the curve (AUC) of 0.58 in the test set. To develop a more powerful predictive tool, a combined model was built by integrating the Radscore with the two selected clinical features. This multimodal dataset was used to train and evaluate three distinct machine learning classifiers: Random Forest, Support Vector Machine, and Logistic Regression. The performance of each classifier was rigorously assessed to identify the optimal integrated model for predicting HER2 overexpression.

**Table 3:** Univariate logistics regression analysis in the training set.

Preoperative baseline characteristics	Univariate logistic regression	
	OR (95% CI)	P-value
Age (per 5 yr)	0.864 (0.676-1.104)	0.243
PSA level (interquartile OR)	1.332 (0.985-1.799)	0.062
Prostate Volume (interquartile OR)	1.042 (0.777-1.397)	0.784
PSAD (interquartile OR)	1.213 (0.901-1.633)	0.203
Lesion Dimension On MRI (interquartile OR)	1.229 (0.914-1.653)	0.172
Positive Biopsy Cores/Total Cores (interquartile OR)	1.039 (0.782-1.382)	0.790
Biopsy ISUP Grade Group (3/4/5 *vs*. 1/2)	1.953 (0.996-3.829)	0.051
PI-RADS Score (5 *vs*. 3/4)	1.056 (0.538-2.074)	0.873
Tumor Focality	0.725 (0.374-1.406)	0.341
Radscore (interquartile OR)	4.422 (2.762-7.082)	<0.001

Univariate Logistic Regression in the training set. PSA, prostate-specific antigen; PSAD, prostate- specific antigen density; MRI, magnetic resonance imaging; ISUP Grade, International Society of Urological Pathology Grade; PI-RADS, Prostate Imaging Reporting and Data System. OR, Odds Ratio; 95% CI, 95% Confidence Interval.

### Model performance and observations

**Figure 5:**
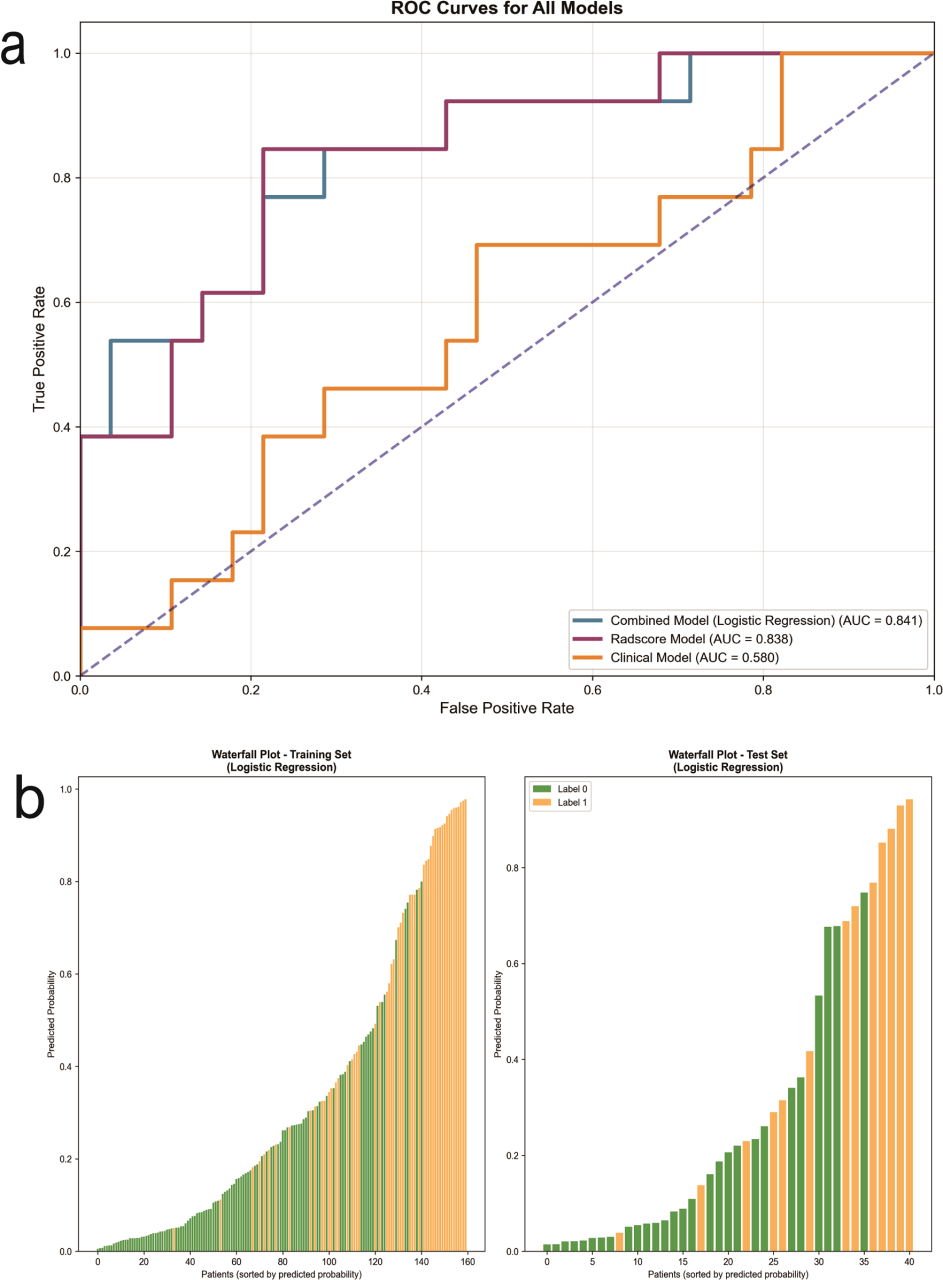
**(A)** Summary graph of AUC curves for the clinical model, radscore model, and combined model constructed by logistic regression on the test set. **(B)** Waterfall plot visualization of the combined model on the training and test set, with green boxes representing no or low HER2 expression and yellow boxes representing HER2 overexpression.

In this study, the Logistic Regression-based combined model (Combined LR) demonstrated superior discriminative performance, achieving the highest AUC of 0.841 (95% CI: 0.697–0.955) alongside an accuracy of 0.780, a sensitivity of 0.769, and a specificity of 0.786 on the test set. Its well-balanced performance across sensitivity and specificity underscores its robustness as a classifier. In contrast, the Clinical model exhibited limited predictive utility, with an AUC of only 0.580 (95% CI: 0.382–0.773), significantly lower than all other models. The Radscore model alone showed strong performance with an AUC of 0.838 (95% CI: 0.693–0.951), markedly outperforming the Clinical model (ΔAUC = 0.258), which highlights the substantial value of radiomic features beyond conventional clinical variables (**Figure 5**). Integration of clinical features with the Rad-score further enhanced predictive ability, as evidenced by the Combined LR model’s slight but meaningful improvement over the Radscore-only model (ΔAUC = 0.003). This suggests complementary information between imaging-derived and clinical predictors. **Table 4** summarizes the performance metrics for all models developed in this study.

**Table 4:** Evaluation of the all the models on the training and test set.

Model	AUC (95%CI)	Accuracy	Sensitivity	Specificity	Precision	F1-Score	NPV
Radscore	0.838 (0.693-0.951)	0.78	0.769	0.786	0.625	0.69	0.88
Clinical	0.580 (0.382-0.773)	0.585	0.692	0.536	0.409	0.514	0.789
Combined RF	0.723 (0.540-0.869)	0.659	0.769	0.607	0.476	0.588	0.85
Combined SVM	0.764 (0.544-0.925)	0.78	0.769	0.786	0.625	0.69	0.88
Combined LR	0.841 (0.697-0.955)	0.78	0.769	0.786	0.625	0.69	0.88

Performance metrics for all developed prediction models. AUC, Area Under the Curve; RF, Random Forest; SVM, Support Vector Machine; LR, Logistic Regression; NPV, Negative Predictive Value.

**Figure 6:**
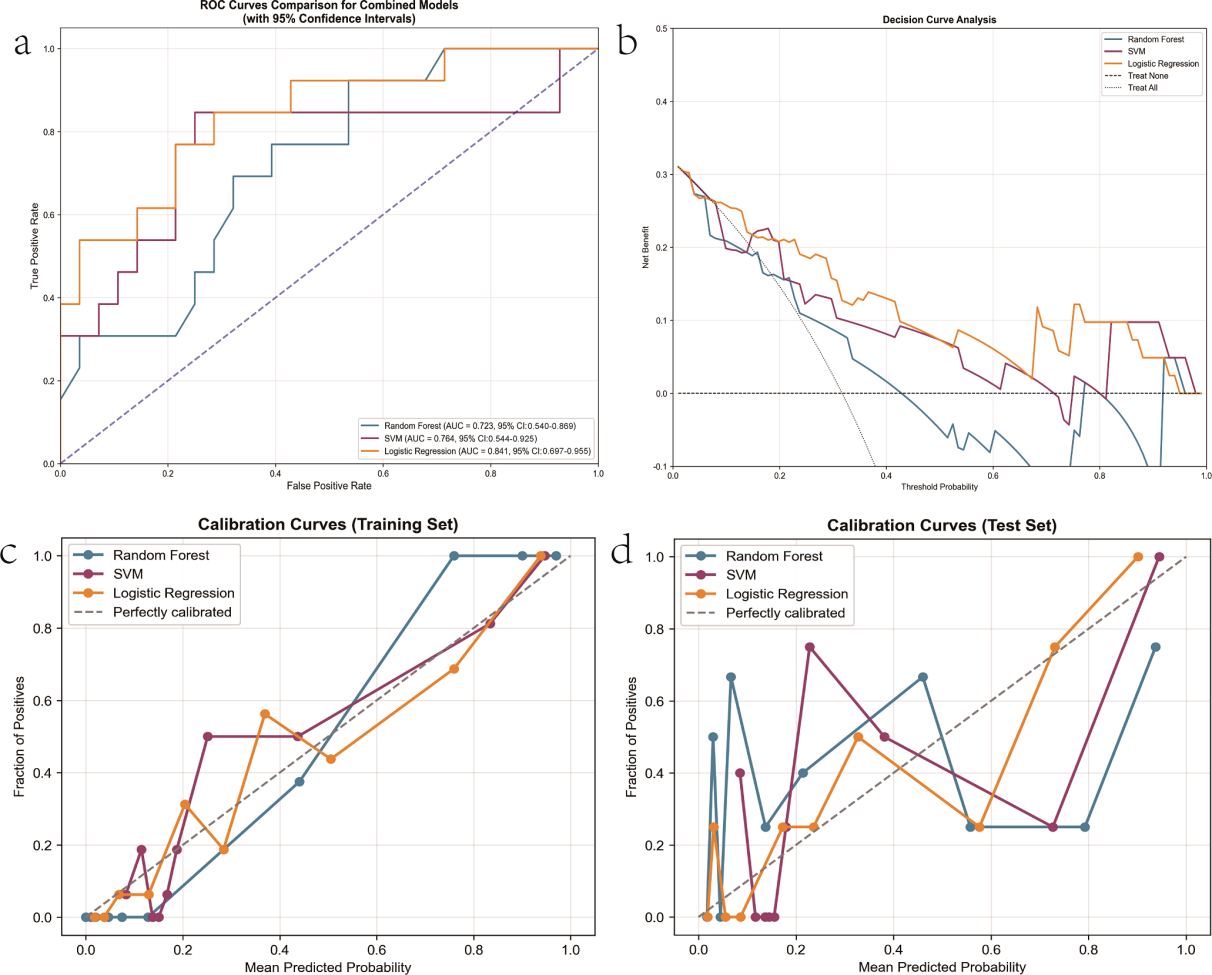
Validation of predictive models using an independent test cohort. The performance of Random Forest (RF), Support Vector Machine (SVM), and Logistic Regression (LR) is shown. **(a)** Receiver Operating Characteristic (ROC) curves with Area Under the Curve (AUC) values (with 95% confidence intervals estimated via bootstrap). **(b)** Decision curve analysis (DCA) comparing the net benefit of the models against default strategies. **(c–d)** Calibration curves for the training and test sets, demonstrating model generalizability.

Among the other combined models, the SVM classifier achieved competitive accuracy and specificity but did not surpass the LR model in overall discriminative power (AUC = 0.764). The RF-based combined model yielded more moderate results (AUC = 0.723), indicating possible underfitting or suboptimal hyperparameter tuning (**Figure 6**). To minimize the variability associated with random data partitioning, we conducted 100 iterations of repeated random sub-sampling validation (Monte Carlo cross-validation). The average performance metrics for the final model over the 1000 random 80/20 splits are as follows: Average AUC: 0.84 ± 0.03; Average Accuracy: 0.77 ± 0.04; Average Sensitivity: 0.60 ± 0.13; Average Specificity: 0.85 ± 0.07. The distribution of the AUC values is presented in **Supplementary Figure S1**, and the results (mean ± standard deviation) are reported in **Supplementary Table T1**. These results confirm that the predictive performance of our modeling strategy is stable and not dependent on a single, potentially favorable data split. In summary, the Combined LR model emerged as the optimal predictor in this task, successfully integrating radiomic and clinical information to achieve the highest diagnostic accuracy. Future work will focus on external validation and interpretability-driven analysis to elucidate feature contributions and facilitate clinical translation.

## Discussion

Our study validates the feasibility of predicting HER2 overexpression in prostate cancer using radiomic features derived from bp-MRI, supporting the broader potential of imaging-based molecular phenotyping in oncology.

Current radiomic research in prostate cancer has primarily targeted the prediction of well-established biomarkers such as TP53 and Ki-67, in addition to conventional clinical endpoints like biochemical recurrence (BCR) and extracapsular extension (EPE), which have been extensively investigated (20, 21). For instance, Ruchuan Chen et al. utilized Random Forest and SVM algorithms to predict TP53 mutations based on peritumoral texture features, achieving AUCs of 0.84 and 0.83 in internal and external validation, respectively. In a similar vein, Xiaofeng Qiao et al. developed a machine learning model incorporating T2-weighted and ADC images to predict both Ki-67 expression and Gleason grade groups (GGG), with reported AUCs of 0.88 and 0.92—significantly outperforming a prior deep learning model for Ki-67 prediction (AUC = 0.75) (22–24). Based on the existing literature, radiomics approaches have demonstrated promising utility in discriminating HER2 expression status in breast cancer, as evidenced by studies from Ramtohul et al. and Zheng et al. (25, 26). These studies successfully developed multiparametric MRI-based radiomics models to differentiate HER2-zero, -low, and -positive breast cancers, achieving AUCs of 0.80 and 0.725-0.889 across different validation cohorts, respectively. Their methodologies, incorporating feature selection techniques such as LASSO regression, align with the analytical framework employed in our current study.

To the best of our knowledge, this study represents the first systematic effort to develop a radiomics-based model for predicting HER2 overexpression specifically in prostate cancer. Against this backdrop, our model demonstrates competitive performance, with an AUC of 0.841 in the test cohort, thereby filling a significant gap in the field of prostate cancer molecular imaging.

Beyond prostate-specific applications, deep learning approaches have demonstrated transformative potential in surgical oncology and molecular imaging. For instance, Sibilano et al. developed a semantic segmentation model for robot-assisted prostatectomy, illustrating how AI can enhance anatomic localization and procedural precision (27). In a related domain, Ali et al. showcased the utility of radiolabeled molecular imaging for rheumatoid arthritis, underscoring the cross-disciplinary potential of quantitative imaging biomarkers for disease characterization and treatment monitoring (28). These studies collectively affirm that imaging-based AI models can capture subtleties beyond human visual assessment. Nevertheless, while deep learning approaches have shown promise in certain domains, their performance in specific radiomic classification tasks—such as EPE prediction—has been modest. A recent meta-analysis reported an AUC of 0.72 for deep learning models compared to 0.82 for leading radiomic models in this context (29, 30).

The successful radiomic prediction of HER2 status holds particular clinical significance in the context of castration-resistant prostate cancer (CRPC), where HER2 overexpression has been implicated in disease progression and resistance to conventional androgen receptor pathway inhibitors. Accumulating evidence suggests that HER2 amplification and overexpression may activate alternative signaling cascades that bypass androgen dependency, thereby sustaining tumor growth in the castration-resistant setting. Our non-invasive prediction approach may thus facilitate improved patient stratification and help guide targeted therapeutic interventions—akin to the established role of HER2-directed therapy in breast cancer. The recent FDA approval of trastuzumab deruxtecan (T-DXd) for HER2-positive solid tumors further underscores the translational relevance of HER2 as a actionable target in advanced prostate cancer. Together with advances in predicting TP53 and Ki-67, our findings underscore the expanding utility of radiomics in elucidating tumor biology and argue for the integration of AI-derived imaging biomarkers into future clinical prognostication frameworks.

## Limitations and future work

However, our study has several limitations that should be considered. First, HER2 overexpression remains relatively uncommon in prostate cancer and has not yet been integrated into routine clinical biomarker assessment. Further validation is required to establish its prognostic and predictive utility, which would enable more accurate risk stratification and personalized treatment selection. Second, this was a retrospective analysis with a limited sample size from a single institution. Future work should prioritize external validation using multi-institutional datasets acquired from different scanner vendors to evaluate model generalizability. Additionally, prospective study designs are needed to minimize selection bias and verify clinical applicability. Importantly, subsequent research should also investigate the correlation between imaging-based HER2 predictions and treatment response in castration-resistant prostate cancer (CRPC) patients receiving HER2-targeted therapies, which would help validate the clinical relevance of our radiomic biomarker. Through these efforts, we hope this line of research will ultimately contribute to more refined, image-guided personalized treatment strategies for prostate cancer patients.

## Conclusion

In this study, we successfully developed and validated an integrated radiomics-clinical model for predicting HER2 overexpression in prostate cancer, demonstrating robust predictive performance. This approach provides a non-invasive framework for HER2 status assessment, paving the way for image-guided molecular phenotyping in oncology. By enabling pre-treatment identification of HER2-overexpressing subtypes, our model holds significant potential to inform personalized therapeutic strategies, particularly in the era of antibody-drug conjugates and other HER2-targeted agents. These findings underscore the evolving role of radiomics as a decision-support tool in precision oncology and highlight its capacity to help tailor treatment selection toward more individualized patient care.

## Data Availability

The raw data supporting the conclusions of this article will be made available by the authors, without undue reservation.

## Supplementary material

Supplementary Figure 1 **(A)** Distribution of AUC across 100 random splits. **(B)** Box plot of AUC across 100 random splits.
