# Supplementary figures and images for: Predicting HER2 overexpression in prostate cancer using machine learning: implications for personalized therapy
Source: Front Oncol. 2026 Jan 13;15:1707946. doi: 10.3389/fonc.2025.1707946 (PMC12834805; doi:10.3389/fonc.2025.1707946)

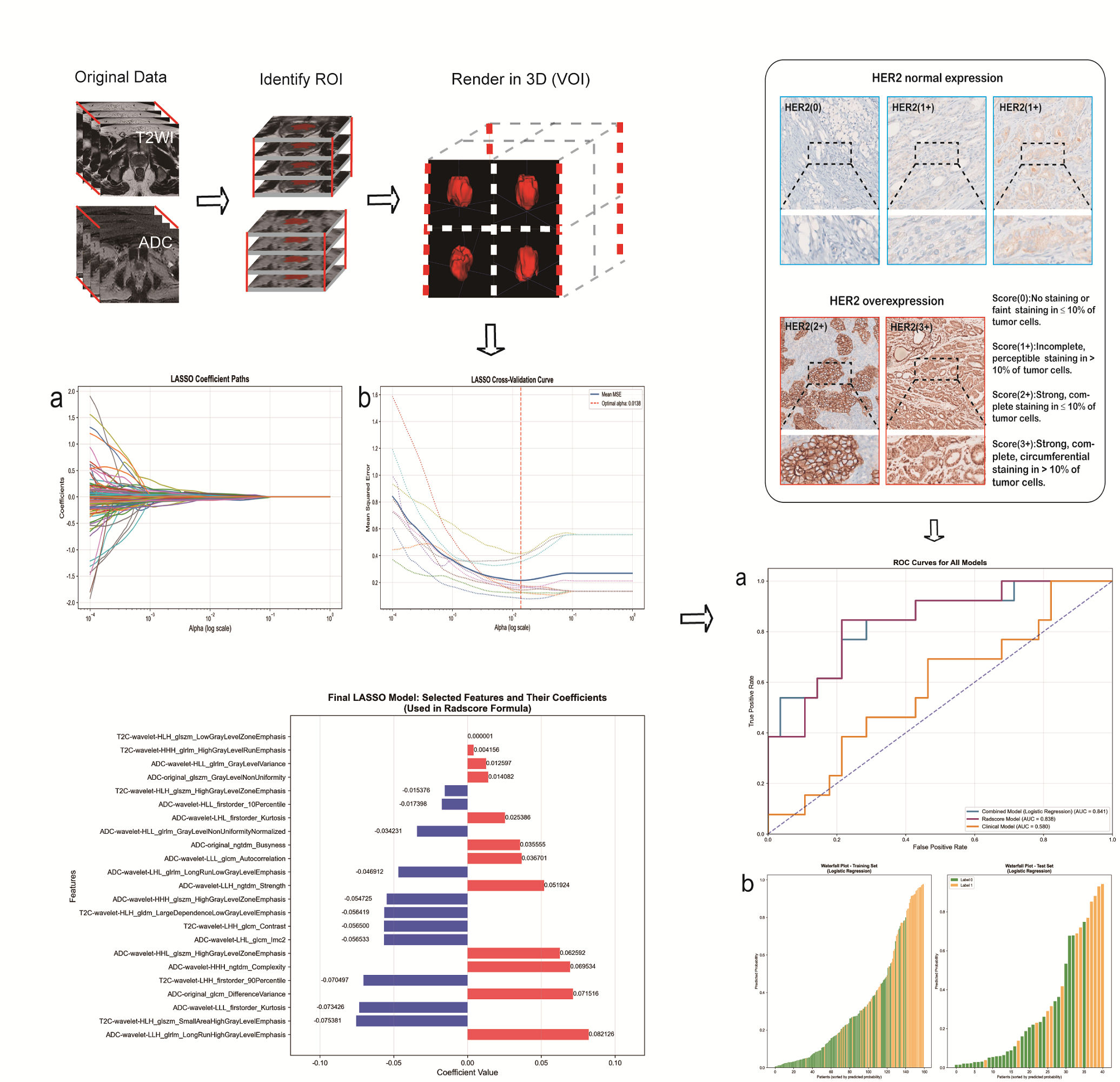

Supplement: Supplementary Figure 1 — (A) Distribution of AUC across 100 random splits. (B) Box plot of AUC across 100 random splits. [file Image1.tif]

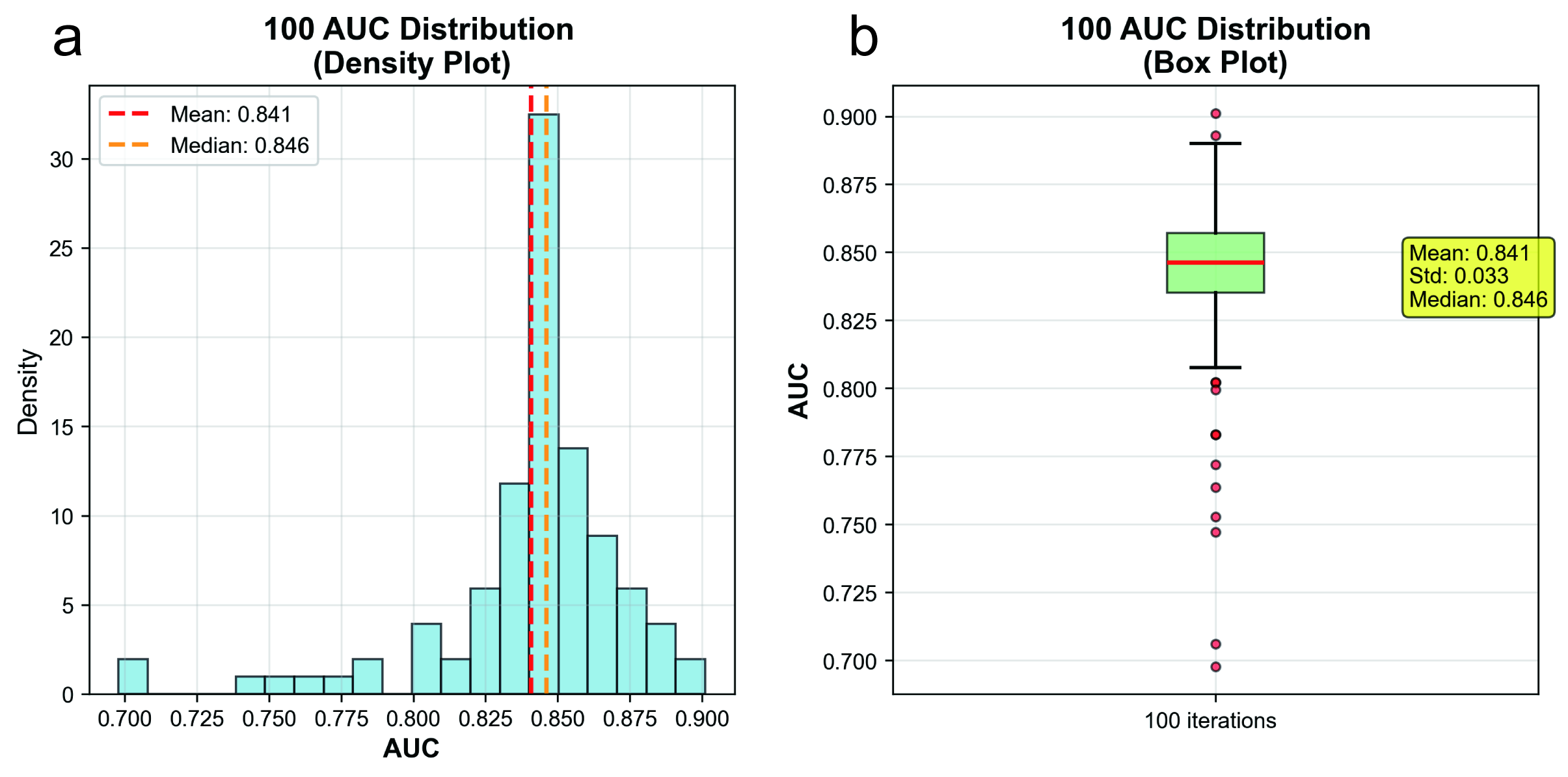

Supplement: Supplementary file 2 [file Image2.tif]
