# Supplementary material for: Predicting HER2 overexpression in prostate cancer using machine learning: implications for personalized therapy
Source: Front Oncol. 2026 Jan 13;15:1707946. doi: 10.3389/fonc.2025.1707946 (PMC12834805; doi:10.3389/fonc.2025.1707946)
Supplement: Supplementary file 3 [file DataSheet1.docx]

Radscore = - 0.070497 × T2C-wavelet-LHH_firstorder_90Percentile + 0.014082 × ADC-original_glszm_GrayLevelNonUniformity - 0.056533 × ADC-wavelet-LHL_glcm_Imc2 - 0.054725 × ADC-wavelet-HHH_glszm_HighGrayLevelZoneEmphasis - 0.017398 × ADC-wavelet-HLL_firstorder_10Percentile + 0.071516 × ADC-original_glcm_DifferenceVariance - 0.034231 × ADC-wavelet-HLL_glrlm_GrayLevelNonUniformityNormalized - 0.073426 × ADC-wavelet-LLL_firstorder_Kurtosis - 0.015376 × T2C-wavelet-HLH_glszm_HighGrayLevelZoneEmphasis + 0.004156 × T2C-wavelet-HHH_glrlm_HighGrayLevelRunEmphasis + 0.012597 × ADC-wavelet-HLL_glrlm_GrayLevelVariance + 0.069534 × ADC-wavelet-HHH_ngtdm_Complexity - 0.056419 × T2C-wavelet-HLH_gldm_LargeDependenceLowGrayLevelEmphasis + 0.051924 × ADC-wavelet-LLH_ngtdm_Strength + 0.036701 × ADC-wavelet-LLL_glcm_Autocorrelation + 0.062592 × ADC-wavelet-HHL_glszm_HighGrayLevelZoneEmphasis + 0.082126 × ADC-wavelet-LLH_glrlm_LongRunHighGrayLevelEmphasis - 0.075381 × T2C-wavelet-HLH_glszm_SmallAreaHighGrayLevelEmphasis - 0.046912 × ADC-wavelet-LHL_glrlm_LongRunLowGrayLevelEmphasis + 0.000001 × T2C-wavelet-HLH_glszm_LowGrayLevelZoneEmphasis + 0.025386 × ADC-wavelet-LHL_firstorder_Kurtosis - 0.056500 × T2C-wavelet-LHH_glcm_Contrast + 0.035555 × ADC-original_ngtdm_Busyness
